# Altered network efficiency of functional brain networks in patients with breast cancer after chemotherapy

**DOI:** 10.18632/oncotarget.22358

**Published:** 2017-11-09

**Authors:** Han Xuan, Chen Gan, Wen Li, Zhonglian Huang, Longsheng Wang, Qianqian Jia, Zhendong Chen, Huaidong Cheng

**Affiliations:** ^1^ Department of Oncology, Cancer and Cognition Laboratory, The Second Affiliated Hospital of Anhui Medical University, Hefei, Anhui, China

**Keywords:** breast cancer, network, cognitive function, MRI, chemotherapy

## Abstract

**Objective:**

To investigate the topological organization of functional brain networks in chemotherapy-treated breast cancer (BC) patients with source memory impairment.

**Methods:**

Twenty-eight patients with BCfollowingchemotherapyand40age-and education-matched healthy controls (HCs) were recruited in the current study. All participants underwent source memory tests and resting-state functional MRI scans. Individual whole-brain functional brain networks were constructed and analyzed using graph-based network approaches.

**Results:**

Compared with the HCs, the BC patients showed lower scores in the source memory tests (*P <* 0.001).Graph-based analyses revealed that the patients showed higher absolute global and local efficiency (both *P* < 0.01) but lower normalized global and normalized local efficiency (both *P*< 0.001) compared with the HCs. Locally, several prefrontal, occipital, and parietal regions exhibited higher nodal efficiency and functional connectivity in the patients(*P*< 0.05, corrected). Finally, positive correlations were observed between normalized global efficiency and Mini-Mental State Examination scores (r = 0.398, *P* = 0.036) and between normalized local efficiency and the source memory scores (r = 0.497, *P* = 0.01) in the patients.

**Conclusion:**

Chemotherapy-treated BC is associated with abnormal organization of large-scale functional brain networks, which could account for source memory dysfunction in patients with BC.

## INTRODUCTION

Chemotherapy-induced cognitive impairments (CICI) involve multiple cognitive domains including attention, memory, executive function, and information processing speed in patients with cancer during or after chemotherapy. Breast cancer (BC) is one of the most prevalent types of cancer, with a higher incidence, but it also has better treatment outcomes than others. Patients with BC can survive for a long time, and chemotherapy has therefore become an important issue with long-term effects on the life quality of patients with BC. In 2014, experts suggested initiating guidelines for assessment and management of chemobrain [1]. JCO also published a new study on chemobrain, which indicated that chemobrain is one of the most important areas of research [2].However, the neural mechanism underlying BC chemobrain is not currently well established, particularly from a system-level network integrity perspective.

Previous neurophysiological studies have documented that patients with BC after chemotherapy have subjective and objective cognitive damage [3, 4]. However, CICI occurs heterogeneously in BC. A meta-analysis showed that CICI in BC is present in language and visual space [5], while Morean et al.[6]found that it mainly appeared in memory, attention, and social cognition. More recently, several studies found that memory impairment is prominent in CICI in BC, but the degree of damage is specific to different memory components [7]. BC chemobrain is mainly associated with impairment of event-based prospective memory (EBPM) in terms of prospective memory(PM), and with source memory in terms of episodic memory. Overall, these studies collectively suggest that different cognitive domains, rather than a single one, are affected by CICI in patients with BC, indicative of an involvement of multiple functional systems in the disease. This makes network-level studies vital for understanding the neural substrates of CICI in patients with BC.

Brain networks can be derived from different modalities of non-invasive neuroimaging techniques *in vivo* [8]. Specifically, resting-state functional MRI (rs-fMRI) is a promising tool for mapping intrinsic brain connectivity network sand has been widely applied to various brain disorders [9]. This technique measures spontaneous brain activity as low-frequency fluctuations in blood oxygen level-dependent (BOLD) signals [10], which exhibit coherent temporal dynamics within and across different neuroanatomical systems. Using this technique, Kesler et al. [11]found that BC chemobrain was related to decreased functional connectivity of the default mode network. On a more global scale, Bruno and colleagues [12]showed that whole-brain functional networks exhibited a reduced normalized clustering coefficient for patients with BC receiving chemotherapy. However, the study found no correlation between cognitive decline and the network alterations.

In the present study, we systematically investigated the topological organization of functional brain networks in28patients with BC after chemotherapy and 40 age- and education-matched healthy controls (HCs).Specifically, individual functional brain networks were constructed by calculating interregional functional connectivity of spontaneous brain time series signals among 90 regions of interest (ROIs).Graph-based network efficiency was then calculated to topologically characterize the resultant networks at both global and nodal levels. BC-related alterations in these network properties were further statistically tested. Finally, a correlation analysis was carried out to examine the relationship between network alterations and cognitive performance in the patients.

## RESULTS

### Between-group differences in demographic and neuropsychological variables

There were no significant differences in age or education between the patient and control groups (both *P* > 0.05). However, the patients had significantly lower scores on the digit span test, the verbal fluency test, the Mini-Mental State Examination (MMSE) scores, and the source memory task compared to the HCs (all *P* < 0.001) (Table 1).

**Table 1:** Demographics and clinical characteristics and cognition of the participants

	BC (n=28)	HCs (n=40)	*P* value
Age(years)	51.46±8.72	50.23±8.15	0.551
Education (years)	9.25±3.22	9.58±3.44	0.695
MMSE	25.89±2.41	28.35±1.46	<0.001
Digit span	6.64±0.91	11.73±2.09	<0.001
Verbal fluency test	5.43±1.07	6.60±1.03	<0.001
Source memory	0.53±0.04	0.75±0.04	<0.001

BC, breast cancer; HCs, healthy controls; MMSE, Mini-Mental State Examination.

### Between-group differences in small-world efficiency

**Figure 1:**
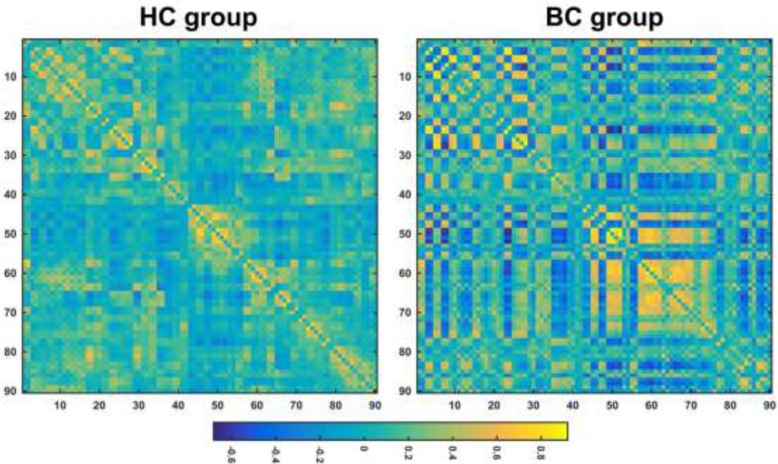
The mean correlation matrices within each group HC, healthy control; BC, breast cancer.

**Figure 2:**
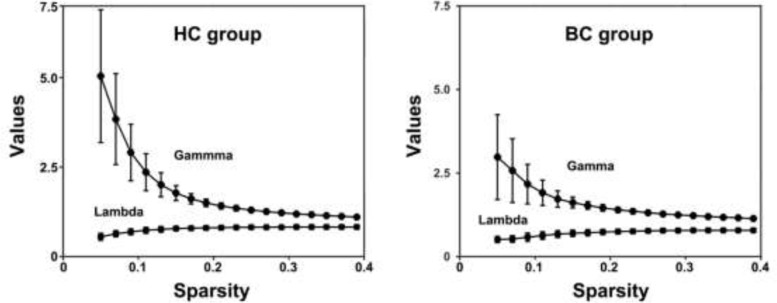
Small-world properties of functional brain networks as a function of sparsity HC, healthy control; BC, breast cancer.

The mean correlation matrices of the BC and HC groups are shown in Figure 1. Both groups exhibited small-world organization of their functional brain networks over the whole sparsity range studied (i.e., normalized global efficiency ∼ 1 and normalized local efficiency > 1) (Figure 2). Further statistical comparisons of the AUCs revealed that the patients showed significantly increased global (*P* = 0.007) and local (*P* < 0.001) efficiency compared with the HCs. However, after the normalization by random networks, these two measures exhibited significantly lower values in the patients compared to the HCs (both *P* < 0.001) (Table 2).

**Table 2:** Between-group differences in global network measures

	HCs (n = 40)	BC (n = 28)	*P* value
Local efficiency^auc^	0.136 ± 0.022	0.166 ± 0.029	<0.001
Global efficiency^auc^	0.084 ± 0.007	0.089 ± 0.008	0.003
Normalized local efficiency^auc^	0.604 ± 0.086	0.527 ± 0.059	<0.001
Normalized global efficiency^auc^	0.265 ± 0.018	0.243 ± 0.022	<0.001

BC, breast cancer; HCs, healthy controls; auc, area under the curve.

We further examined the effect of imbalanced sample numbers between the BC and HC groups (i.e., 28 vs. 40) on the above results. First, we randomly chose 28 HCs from the control group, and computed and recorded the difference in the mean of each network measure between all the BC patients and the subset of HCs. This procedure was iteratively performed 10,000 times to obtain a distribution, which was used to determine whether the between-group network differences derived from all the participants (i.e., 28 BC patients vs. 40 HCs) fell outside its 95% confidence intervals. The results showed that between-group differences of all network derived from all the participants fell within the 95% confidence intervals of the corresponding distributions derived from the balanced sample numbers. Further, we statistically compared network efficiency of the BC patients with each subset of HCs (permutation test), and found significant (*P*< 0.05) between-group differences in most cases (10,000 out of 10,000 for local efficiency, 9,936 out of 10,000 for global efficiency, 10,000 out of 10,000 for normalized local efficiency, and 10,000 out of 10,000 for normalized global efficiency) with the same altered directions to those derived from all the participants. These findings indicate limited effect of imbalanced sample numbers between the BC and HC groups on the above results.

### Between-group differences in nodal efficiency

**Figure 3:**
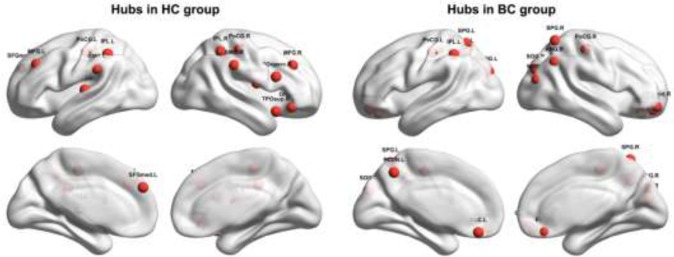
Hubs within each group HC, healthy control; BC, breast cancer. Refer to Table 3 for regional abbreviations.

Figure 3 shows spatial distribution of hubs for each group. In the HC group, we identified 14 hub regions, including 10 association cortex regions, two paralimbic cortex regions and two primary cortex regions. These hubs were mainly located in frontal/prefrontal (the bilateral middle frontal gyri, the opercular part of the right inferior frontal gyrus, the orbital part of the right inferior frontal gyrus, the right rolandic operculum, and the left medial superior frontal gyrus), parietal (the bilateral postcentral gyri, the bilateral inferior parietal, but the supramarginal and angular gyri and the bilateral supramarginal gyri), and temporal (the left superior temporal gyrus and the right temporal pole: superior temporal gyrus) regions. In the BC group, we identified fifteen hub regions, including eight association cortex regions, five paralimbic cortex regions, and two primary cortex regions. These hubs were predominantly located in parietal (the bilateral postcentral gyri, the bilateral superior parietal gyri, the left inferior parietal, but the supramarginal and angular gyri, the right angular gyrus, and the left precuneus), prefrontal (the orbital part of the bilateral superior frontal gyrus, the orbital part of the right middle frontal gyrus, and the bilateral gyrus rectus), and occipital (the bilateral superior occipital gyri and the right middle occipital gyrus) regions. Notably, only three regions were commonly identified as hubs in both groups, including the bilateral postcentral gyrus and the left inferior parietal, but the supramarginal and angular gyri).

**Figure 4:**
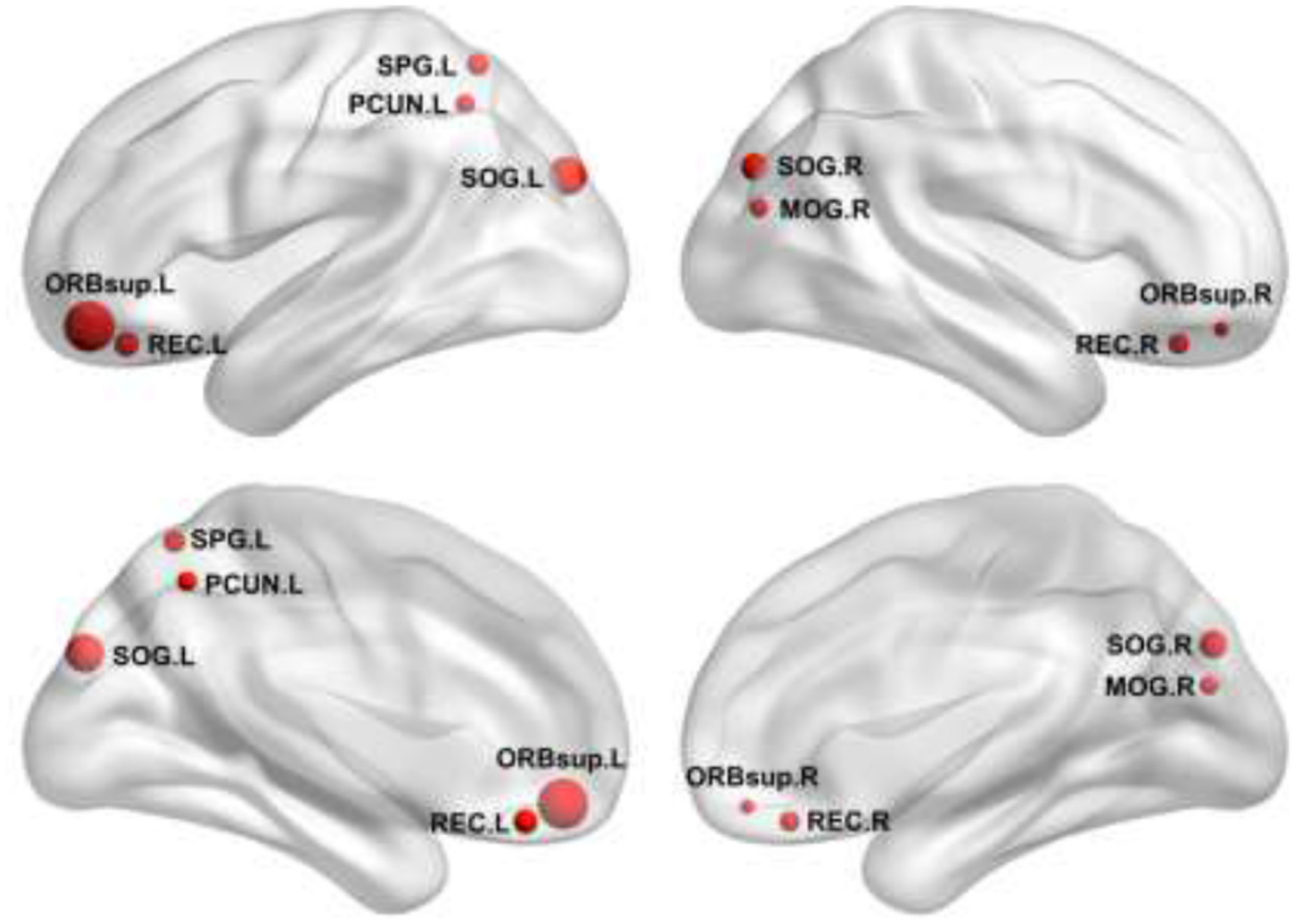
Increased nodal efficiency in the patients Refer to Table 3 for regional abbreviations.

Further between-group comparisons revealed that nine regions showed increased nodal efficiency in the patients with BC compared to the HCs(Figure 4). The regions included the orbital part of the bilateral superior frontal gyrus, the bilateral gyrus rectus, and the bilateral superior occipital gyrus, the left superior parietal gyrus and the left precuneus, and the right middle occipital gyrus. Notably, all these regions were identified as hubs in the patient group. There were no regions showing decreased nodal efficiency in the patients compared to the HCs.

### Between-group differences in functional connectivity

**Figure 5:**
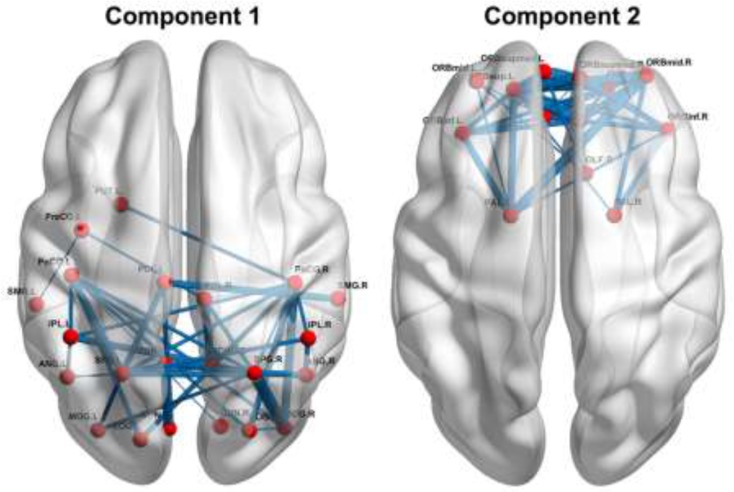
Increased functional connectivity in the patients Refer to Table 3 for regional abbreviations.

We identified two connected components that exhibited increased functional connectivity in the patients with BC compared with the HCs (*P*< 0.001, corrected) (Figure 5). The first component included 22 nodes and 42 edges that were mainly situated in parietal and occipital regions. The other included 13 nodes and 37 edges that were predominantly located in prefrontal regions. It is worth mentioning that all the regions showing increased nodal efficiency described above were included in these two components. No components were found to show significantly decreased functional connectivity in the patients.

### Relationship between network measures and neuropsychological variables

**Figure 6:**
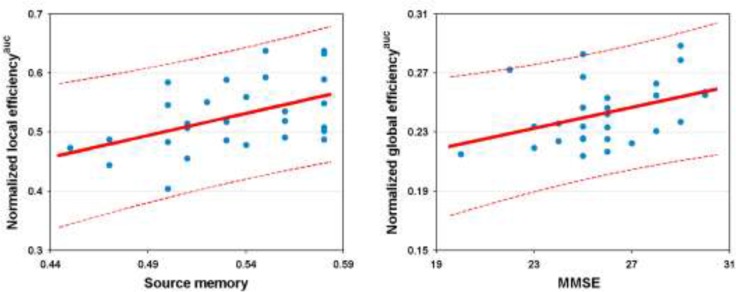
Relationship between network efficiency and cognition in the patients AUC, area under curve; MMSE, Mini-Mental State Examination.

Significantly positive correlations were observed between normalized global efficiency and the MMSE scores (r = 0.398, *P* = 0.036) and between normalized local efficiency and the source memory scores (r = 0.497,*P* = 0.01) in the patients (Figure 6). Of note, these correlations did not survive after correcting for multiple comparisons (*P*> 0.05, corrected by the False Discovery Rate procedure). No significant correlations were found between other network/connectivity measures and cognitive variables(all *P* > 0.05).

## DISCUSSION

The current study investigated topological organization of functional brain networks of chemotherapy-treated patients with BC by combining rs-fMRI and graph theory methods. We observed that compared with the HCs, the patients had cognitive dysfunction in general cognition, short-term memory, executive function, episodic memory, and prospective memory. Further network analyses revealed that the patients showed abnormal network organization of parallel network efficiency in their brain networks. Moreover, the altered network efficiency was associated with clinical variables of the patients. Locally, several prefrontal, occipital, and parietal regions showed increased nodal efficiency and functional connectivity in the patients. Overall, these findings provide empirical evidence for network dysfunction in survivors of BC that may contribute to disturbances of cognitive functions in these patients.

We found that the patients showed cognitive obstacles in multiple cognitive domains including language, memory, and other cognitive functions. This is consistent with previous studies [13, 14]. Particularly, memory impairment seems to be one of the most prominent features of BC chemobrain. Based on the same cohort of patients, these findings confirm that rather than affecting a single domain, BC chemobrain is related to cognitive dysfunction in multiple domains.

Graph-based network analyses of the human brain are becoming popular in neuroscience, because they can provide valuable information for our understanding of how the wiring diagram of the brain might promote information transfer and processing [15]. Moreover, this analytical framework is becoming more and more popular in atypical populations, because accumulating evidence indicates that many neuropsychiatric diseases are associated with abnormal coordination between regions, indicative of network dysfunction. Here, we applied a graph-based network analysis to BC chemobrain and discovered that although functional brain networks of both groups exhibited small-world organization, absolute global efficiency and local efficiency were significantly increased in the BC group compared to the HC group. Global efficiency reflects integrative information processing between and across remote regions of the brain and is mainly associated with long-range connections. Local efficiency reflects modular information processing or fault-tolerance of a network and is predominantly associated with short-range connections between nearby regions. The increase in absolute global and local efficiency thus suggests that the functional brain networks of the patients are more efficient in favor of information propagation, exchange, and processing at both global and local levels. This is consistent with our subsequent analyses of nodal efficiency and functional connectivity, which revealed increased nodal efficiency and interregional coordination of multiple regions in the patients (see below for further discussions on these increases).However, after normalization by random networks, functional brain networks of the patients showed lower normalized global and normalized local efficiency than the HCs. Normalized network efficiency quantifies the deviation or optimization degree of an actual network from matched random networks that have the same number of nodes and edges and the same degree distribution as the actual brain networks. This normalization procedure is important because it corrects for potential differences in network features between groups, such as degree distribution. Indeed, we found that random networks derived from the patient group had significantly higher network efficiency (global and local) than those derived from the HC group (*P*< 0.001). This explains why opposite patterns were observed between absolute and normalized network efficiency in revealing BC-related alterations. The opposite patterns are also consistent with our view that absolute efficiency and normalized network efficiency complement each other to fully characterize topological organization of brain networks. Given that the small-world organization reflects an optimal balance between local specialization and global integration, our findings imply a break of the normal balance in BC. This is consistent with a previous study that showed lower normalized clustering coefficients in functional brain networks of patients with BC [12]. Interestingly, the altered network efficiency was related to cognitive impairments in the patients as evidenced by positive correlations between normalized local efficiency and source memory and between normalized global efficiency and the MMSE scores. That is, the lower the normalized network efficiency of the patient's brain, the worse the cognitive performance of the patient. This suggests a neurocognitive significance of small-world network efficiency in monitoring cognitive impairments in patients with BC. It should be noted that the brain-behavior relationships were not corrected for multiple comparisons, and thus the correlations should be explained with cautions. Future studies are warranted to systematically investigate the associations between brain network alterations and cognitive disturbances in BC.

In addition to topological characterization of the whole brain network, we also investigated regional nodal efficiency and interregional functional connectivity. Nodal efficiency measures the extent of information exchange between a given node and all other nodes in a network and therefore reflects the importance or information load of the node [16]. We found that functional brain hubs were mainly located in association and paralimbic cortex regions of prefrontal and parietal lobes for both groups. Most of these regions have been identified as hubs in previous morphological, structural, and functional brain networks [17–23]. Nevertheless, only three hubs were the same between the two groups, suggesting a remarkable redistribution of functional brain hubs in patients with BC. Further, we found that several regions of the frontal, occipital, and parietal lobes exhibited increased nodal efficiency in the patients. These regions were also found to have increased functional connectivity in the patients. Studies employing voxel-based morphometry [24–26]and multimodal MRI techniques [27]found that patients with BC showed decreased gray matter and white matter volume after chemotherapy in the prefrontal cortex, the posterior parietal cortex, the parahippocampal gyrus, the thalamus, the cingulate, and the precuneus. These findings have shown that brain structural changes caused by chemotherapy are mainly inparietal, frontal, and occipital regions [24–26, 28–30]. These brain regions are consistent with those showing increased nodal efficiency and functional connectivity in the patients in the present study. Because it is highly plastic, the brain can adaptively reorganize to maintain normal function by adjusting regional connectivity profiles when some neurons are inefficient or damaged [31]. The increased nodal efficiency and functional connectivity found in the present study thus suggests that the patients’ brains add or establish new connections to compensatefor destructive effects caused by the disease. The compensatory increases of functional connectivity further lead to increased absolute network efficiency in the patients as discussed above. Indeed, by summarizing findings from 126 network studies, a recent study showed that hyperconnectivity is a common response of the brain to neurological disruption [32]. One possible reason for the hyperconnectivity is that neurological disruption requires ongoing recruitment of available detour paths, and the hyperconnectivity may be optimally expressed by increasing connections through the most central and metabolically efficient regions [33]. This is consistent with our findings that all the regions showing increased nodal efficiency in the patients were hubs. Nevertheless, the biological mechanism accounting for the observed hyperconnectivity is not clear particularly given relatively few network studies in BC. Future studies may provide deeper insights into this issue by combining multimodal imaging methods (e.g., arterial spin labeling and positron emission tomography) and biochemical techniques. Notably, using a seed-based approach, only decreased functional connectivity was found with the anterior cingulate cortex for BC patients with chemotherapy in a previous study [34]. This discrepancy could be attributable to remarkable differences in the interval between the last chemotherapy and subsequent MRI scan (i.e., with in1 month vs. 36.6 ± 4.4 months) and/or different analytical approaches (e.g., regional level vs. voxel level).

This study has several limitations. First, the sample size is relative small for the current pilot study, which could challenge the generality of the current findings and limit the power to detect more subtle effects. For example, our correlation results did not survive after multiple comparison correction. Moreover, stage IV patients with BC were included in the current study. This may further increase the heterogeneity of the disease status and the pathogenesis among the patients, and thus confound the current findings. Future studies are needed to examine BC-related functional brain network alterations and their associations with cognitive disturbances of the patients by recruiting more clinically homogeneous samples. Second, this study is a cross-sectional study. It is difficult to exclude the possibility that brain networks are heterogeneous before chemotherapy. So far there is no literature reporting on the inherent heterogeneity of brain networks in patients with BC. Future studies may help explain the current findings by investigating brain network dysfunctions before chemotherapy and longitudinal network changes between pre-chemotherapy and post-chemotherapy. Third, the human brain is a complex system with many non-trivial organizational principles. In addition to efficient small-world organization, future studies need to examine other network features in patients with BC, such as rich-club organization, modular structures, and dynamic network evolution. Fourth, this study only explores functional brain networks of CICI in BC. Although accumulating evidence suggests a strong relationship between structural and functional connectivity [35, 36], there is a lack of one-to-one correspondence [37]. Moreover, different topological features are observed between structural and functional brain networks [38]. Thus, a fusion analysis of multimodal neuroimaging data is needed in the future to examine similarities and differences between structural and functional brain networks, to reveal BC-related topological abnormalities. Finally, we note that the patients included in this study are less well-educated than those in previous studies. It is interesting to investigate whether and how BC-related functional brain network alterations depend on different levels of education and thus cognitive reserve of the patients.

In summary, this study provides evidence for brain dysfunction in survivors of BC from the viewpoint of the global organization of brain functional networks, by using rs-fMRI. In addition, we also report for the first time that the brain network changes are correlated to the source memory disorder, which may provide novel implications for the possible mechanisms underlying CICI in BC.

## MATERIALS AND METHODS

### Participants

This study is a case-control study. It included 28 patients with breast cancer who underwent postoperative adjuvant chemotherapy in the Department of Oncology of the Affiliated Second Hospital of Anhui Medical University in China from January 2014 to June 2015. Neuropsychological tests and MRI scans were performed one month after the last chemotherapy for the patients. Forty age-matched and education-matched adults (mainly relatives of the breast cancer patients and workers of the same hospital) were recruited through advertisements as control subjects. All subjects were right-handed. All patients were at BC stages I–IV and were treated with six cycles of standard dose TEC chemotherapy (docetaxel 75 mg/m^2^ intravenous drip d_1_ (doxorubicin 50 mg/m^2^ intravenous drip d_1_; cyclophosphamide 500 mg/m^2^ intravenous drip d_1_; 21 days per cycle). The pathology of all patients was infiltrating ductal carcinoma, ECOG 0-1. Chemotherapy regimens and doses for all patients were in accordance with NCCN guidelines. The exclusion criteria included the following: (1) central nervous system radiation or intrathecal therapy; (2) current or past alcohol or drug dependence; (3) neurobehavioral risk factors including neurologic, medical, or psychiatric conditions known to affect brain structure or function, except a history of depression or anxiety; (4) any MRI contraindications; (5) patients with brain metastases; (6) patients with obvious psychological distress and fatigue in terms of the Distress Thermometer and National Cancer Institute Common Toxicity Criteria. The MRI data of all patients were collected within one month after six cycles of chemotherapy. This study was approved by the Research Ethics Review Board of the Second Affiliated Hospital of Anhui Medical University, and written informed consent was obtained from each participant.

### Neuropsychological tests

The following neuropsychological tests were administered to all participants: (1) the MMSE to measure global cognitive functions, (2) the verbal fluency (number of animals per minute) test to measure frontal-temporal functions, and (3) the digit span test to estimate short-term memory and executive function, including forward and backward spans.

### Source memory tests

#### Materials

There were eight categories of daily-life common objects (fruits, animals, clothing, tools, furniture, transportation tools, stationery and household electrical appliances). Two objects were selected from each category; one was presented with a Jane diagram (selected images from Snodgrass), while the other was presented with content words.

#### Procedure

(1) Learning phase: Subjects were visually presented with one content word and one real figure from each category. The participants were then asked to choose another object of the same category and remember it. There were a total of 24 things from eight categories. (2) Testing phase: After 5 minutes of studying, the subjects were randomly presented with48 content words that belonged to eight categories. Of these 48 content words,24 items were learned (content words, real figures, or associated objects). The other 24 objects were interferential. Subjects were asked to make a new (interferential object) or old (learned object) judgment in of the recognition task. Subjects were then asked to make a judgment of the source memory task from the learned object. In addition, subjects were asked to indicate the order of the project presentation in the learning phase (content words, real figures, or associated objects) [39].

#### Source memory performance

Performance was calculated as correct numbers/total target numbers. All tests were completed by one doctor to eliminate bias.

### MRI image acquisition and preprocessing

All MR images were acquired using a Siemens Verio 3.0 T scanner (Siemens, Erlangen, Germany) at the Second Affiliated Hospital Cancer Institute of Anhui Medical University. Functional images were collected using an echo-planar imaging (EPI) sequence with the following acquisition parameters:32 axial slices; repetition time (TR) = 2000ms; echo time (TE) = 30 ms; flip angle (FA) = 9°; slice thickness = 4 mm; no gap; matrix = 64×64; and field of view (FOV) = 220×220 mm^2^. During the scanning, all participants were instructed to keep their eyes closed, relax their minds, and remain as motionless as possible. The scan lasted for 480 s. Additionally, individual structural images were also acquired for the registration purpose using a T1-weighted gradient echo spiral pulse sequence: 192 axial slices; TR = 1700 ms; TE = 2.98 ms; FA = 9°; slice thickness = 1.0 mm; no gap; matrix = 256×256; and FOV = 256×256 mm^2^.

All functional data preprocessing, network construction, topological analysis, and statistical comparisons were performed using the GRETNA toolbox [40] based on SPM 8 (http://www.fil.ion.ucl.ac.uk/spm/software/spm8/). First, the first five volumes were removed for each participant to allow for T1 equilibration effects. The remaining images were then corrected for intra-volume time offsets between slices (Sinc interpolation) and inter-volume geometrical displacements due to head motion (six-parameter rigid-body transformation). No participants were excluded based on the criterion of a displacement > 2.5 mm or an angular rotation > 2.5 degree in any direction. There were no significant differences in summary measures of head motion profiles between the patients and controls, including the maximum, root mean square and mean frame-wise displacement (all *P* > 0.05). Subsequently, all corrected functional images were normalized to the Montreal Neurological Institute (MNI) space using the transformation fields that were derived from tissue segmentation of individual structural images and were resampled to 3-mm isotropic voxels. The normalized images further underwent removal of linear drifts and temporal band-pass filtering (0.01 - 0.08 Hz) to reduce the effects of low-frequency drift and high-frequency physiological noises. Finally, several nuisance signals were regressed out from each voxel's time series to exclude non-neuronal sources, including 24-parameter head motion profiles, white matter signals, cerebrospinal fluid signals and global signals. The whiter matter, cerebrospinal fluid and global signals were derived by averaging signals within white matter, cerebrospinal fluid and whole-brain masks, respectively, in terms of prior probability maps and brain mask file in SPM8 (threshold = 0.8).

### Network construction and analysis

#### Correlation matrix

In the current study, we calculated interregional functional connectivity matrix for each participant at a macroscopic level. First, the cerebrum was divided into 90 ROIs (45 for each hemisphere; Table 3 ) in terms of a prior Anatomic Automatic Labeling atlas [41]. The Anatomic Automatic Labeling atlas is one of the most commonly used one in previous brain network studies [42]. Then, the mean time series was extracted for each ROI by averaging the time series of all voxels within it, therefore resulting in 90 regional mean time series for each participant. The resultant regional mean time series were subsequently correlated (Pearson correlation) with each other and finally generated a 90 × 90 correlation matrix for each participant. The correlation matrices could be modeled as graphs and further topologically characterized by graph theory-based network approaches. In graph theory, a network or graph is composed of nodes and edges, with nodes representing ROIs and edges representing interregional functional connectivity in the current study. Notably, before subsequent network analyses, negative correlations were excluded (set to 0) in all correlation matrices given their ambiguous interpretation [43–45]and distinct connectivity patterns [46].

**Table 3:** Regions of interest

Index	Regions	Abbreviations	Index	Regions	Abbreviations
1,2	Superior frontal gyrus, dorsolateral	SFGdor	47,48	Middle frontal gyrus, orbital part	ORBmid
3,4	Middle frontal gyrus	MFG	49,50	Inferior frontal gyrus, orbital part	ORBinf
5,6	Inferior frontal gyrus, opercular part	IFGoperc	51,52	Superior frontal gyrus, medial orbital	ORBsupmed
7,8	Inferior frontal gyrus, triangular part	IFGtriang	53,54	Gyrus rectus	REC
9, 10	Rolandic operculum	ROL	55,56	Insula	INS
11,12	Supplementary motor area	SMA	57,58	Anterior cingulate and paracingulate gyri	ACG
13,14	Superior frontal gyrus, medial	SFGmed	59,60	Median cingulate and paracingulate gyri	DCG
15,16	Cuneus	CUN	61,62	Posterior cingulate gyrus	PCG
17,18	Lingual gyrus	LING	63,64	Parahippocampal gyrus	PHG
19,20	Superior occipital gyrus	SOG	65,66	Temporal pole: superior temporal gyrus	TPOsup
21,22	Middle occipital gyrus	MOG	67,68	Temporal pole: middle temporal gyrus	TPOmid
23,24	Inferior occipital gyrus	IOG	69,70	Olfactory cortex	OLF
25,26	Fusiform gyrus	FFG	71,72	Hippocampus	HIP
27,28	Superior parietal gyrus	SPG	73,74	Amygdala	AMYG
29,30	Inferior parietal, but supramarginal and angular gyri	IPL	75,76	Caudate nucleus	CAU
31,32	Supramarginal gyrus	SMG	77,78	Lenticular nucleus, putamen	PUT
33,34	Angular gyrus	ANG	79,80	Lenticular nucleus, pallidum	PAL
35,36	Precuneus	PCUN	81,82	Thalamus	THA
37,38	Paracentral lobule	PCL	83,84	Precental gyrus	PreCG
39,40	Superior temporal gyrus	STG	85,86	Calcarine fissure and surrounding cortex	CAL
41,42	Middle temporal gyrus	MTG	87,88	Postcentral gyrus	PoCG
43,44	Inferior temporal gyrus	ITG	89,90	Heschl gyrus	HES
45,46	Superior frontal gyrus, orbital part	ORBsup			

Regions of left and right hemispheres are indexed by odd and even numbers, respectively.

#### Threshold selection

Before the graph theory-based network analysis of the correlation matrices derived above, a thresholding procedure was first used to convert them into binary networks, whose elements were either 0 or 1, indicating the absence or presence of an edge between two nodes, respectively. This was achieved by applying a correlation coefficient threshold to individual correlation matrices such that correlation coefficients greater than the threshold were set to 1, and others were set to 0. However, if the same correlation coefficient threshold was applied to all individual correlation matrices, the resultant networks will have different numbers of edges across participants and particularly between the two groups due to differences in overall connectivity strength of the correlation matrices. This could confound subsequent between-group comparisons of network topology [9, 47]. To address this concern, a subject-specific correlation threshold was used in the current study to ensure that all resultant networks have the same number of edges and network cost by assigning a fixed sparisty, *S*, which is defined as the ratio of the number of actual edges divided by the maximum possible number of edges in a network. For example, for a given sparsity *S* = 0.1, only the strongest 10% correlation coefficients were retained and set to 1 for each individual correlation matrix. The sparsity-based thresholding procedure therefore allows examining network organization after ruling out confounding effect of different network costs between groups. However, given the lack of a canonical way to determine a single sparsity, we repeatedly thresholded each correlation matrix to generate binary networks with different sparsity values in a continuous range of 0.05 <*S*< 0.4 (interval = 0.02). This enabled us to characterize network organization as a function of sparsity and thus minimize potential bias introduced by a precise selection of single sparsity. The inferior limit of the sparsity was determined to guarantee that the resultant networks were estimable for small-worldness [48] and the superior limit was empirically chosen to ensure that resultant networks had sparse properties [16]. Subsequently, we studied the small-world organization and nodal efficiency for all brain networks at each sparsity threshold.

#### Small-world efficiency

In the current study, we employed both abo efficiency metric (local efficiency and global efficiency) to characterize economical small-world properties of functional brain networks derived above. Compared with conventional small-world parameters of clustering coefficient and characteristic path length [48], the efficiency metric is biologically more sensible and has a number of technical and conceptual advantages. For example, it can represent how efficiently a network exchanges information at local and global levels with a single measure, and deal with either the disconnected or nonsparse graphs or both [16, 49, 50]. Moreover, the efficiency metric has been widely used to study the small-world behavior in previous brain network studies under both normal and pathological conditions [16, 42, 51]. Mathematically, the global efficiency of a network *G* with N nodes is defined as [50]:

$$E_{glob}\left(G\right)=\frac{1}{N(N-1)}\underset{i\neq j\in G}{\sum }\frac{1}{d_{ij}},$$

where *d_ij_* is the shortest path length between node *i* and node*j* and is calculated as the smallest number of edges among all possible paths from node *i* to node *j*. The global efficiency measures the ability of parallel information transfer over the entire network. The local efficiency of *G* is calculated as [50]:

$$E_{loc}(G)=\frac{1}{N}\underset{i\in G}{\sum }E_{glob}(G_{i}),$$

where *E_glob_*(*G_i_*) is the global efficiency of *G_i_*, the subgraph composed of the neighbors of node *i* (i.e., nodes linked directly to node *i*). The local efficiency reflects how well the network exchanges information locally or how much the network is fault tolerant.

To determine whether the brain networks were organized in a small-world manner, the global and local efficiency of each participant derived at each sparsity level were normalized by dividing them by the corresponding mean of 100 random networks. The random networks were generated using a topological rewiring algorithm that preserved the same number of nodes, edges and degree distributions as the real brain networks [52, 53]. Typically, a network with approximately equal global efficiency and larger local efficiency (i.e., normalized global efficiency or λ ∼ 1 and normalized local efficiency or γ > 1) than matched random networks is said to be small-world [48].

#### Nodal efficiency

For each node, we calculated the nodal efficiency to capture their roles in the brain network. Specifically, for a given node *i* in the network *G*, the nodal efficiency is defined as the average shortest path length between node *i* and all other nodes in the network [16]:

$$e_{i}=\frac{1}{N-1}\underset{j\neq i\in G}{\sum }\frac{1}{d_{ij}},$$

where *d_ij_* is the shortest path length between node *i* and node*j* in *G*. Nodal efficiency reflects the ability of a node to exchange information with the rest of the nodes in the network. Regions with high nodal efficiency are typically considered hubs in the brain. In the current study, regions with nodal efficiency at least one standard deviation above the group mean (across all nodes and participants) were identified as hubs within each group.

### Statistical analysis

#### Between-group differences

For demographic and clinical variables, between-group differences were examined with two-sample, two-tailed t-tests; these variables included age, education, digit span, verbal fluency test, source memory, item memory, MMSE, retrospective memory(RM) and prospective memory(PM). For network topological measures (global efficiency, local efficiency, normalized global efficiency, normalized local efficiency, and nodal efficiency), we computed their areas under the curve (AUCs, i.e., the integral over the whole sparsity range), which were used to simplify statistical analysis. Specifically, a non-parametric permutation test was used to examine between-group differences in these network measures. In brief, for each metric, we initially calculated the between-group difference in mean values. An empirical distribution of the difference was then obtained by randomly reallocating all values to two groups and re-computing the mean differences between the two randomized groups (10,000 permutations). The 95th percentile points of the empirical distribution were used as critical values in a one-tailed test to determine whether the observed real-group differences occurred by chance. To examine between-group differences in interregional functional connectivity, a network-based statistic (NBS) method[54] was performed. Briefly, a primary threshold (*P*< 0.01) was applied to the t values (90 × 90 matrix) derived from an edge-by-edge between-group comparison of interregional functional connectivity (two-sample t-test). Among the resultant suprathreshold connections, we identified all connected components and recorded their size (i.e., number of links). To estimate the significance of each identified component, a null distribution of the connected component size was empirically derived by using a permutation approach (10,000 permutations). For each permutation, all subjects were randomly rearranged into two groups, and the same primary threshold (i.e., *P*< 0.01) was used to filter suprathreshold links in the comparison between the two randomized groups. The size of the maximal connected component among these links was recorded to form the null distribution. Finally, for any connected component of size M that was observed in the comparison of the right grouping, the corrected *P* value was determined by calculating the proportion of the 10,000 permutations for which the maximal connected component was larger than M. Notably, only connections that were positive in > 80% of all participants were included in the NBS analysis. Hubs, regions showing abnormal nodal efficiency (*P* < 0.05, Bonferroni corrected), and NBS components showing altered functional connectivity in the patients were visualized on the brain surface using the BrainNet viewer [55].

#### Relationships between network metrics and cognition

Pearson correlation analysis was used to study the relationships between network metrics/functional connectivity showing significant between-group differences and cognitive variables in the patient group.

## Abbreviations

BC: Breast cancer
HCs: Healthy controls
rs-fMRI: Resting-state functional Magnetic resonance image
MMSE: Mini-Mental State Examination
CICI: chemotherapy-induced cognitive impairments
EBPM: event-based prospective memory
RM: retrospectivememory
PM: prospective memory
ROIs: regions of interest
AAL: Automated Anatomical Labelling
BOLD: blood oxygen level-dependent
AUC: area under curve
